# Investigating Advanced Approaches Based on Iso-Absorptivity Coefficient in Unresolved Spectral Signals of Binary Mixtures

**DOI:** 10.1155/2019/7924821

**Published:** 2019-02-03

**Authors:** Hayam M. Lotfy, Sarah S. Saleh

**Affiliations:** ^1^Analytical Chemistry Department, Faculty of Pharmacy, Cairo University, Kasr-El Aini Street, 11562 Cairo, Egypt; ^2^Pharmaceutical Chemistry Department, Faculty of Pharmaceutical Sciences & Pharmaceutical Industries, Future University in Egypt, 12311 Cairo, Egypt; ^3^Analytical Chemistry Department, Faculty of Pharmacy, October University for Modern Sciences and Arts (MSA), 11787 6th of October, Egypt

## Abstract

Several spectrophotometric approaches utilize different functions of the iso-absorptivity coefficient in zero-order absorption signals and its manipulated spectra. This work introduced an investigation concerning the efficiency power of recent methods based on iso-absorptivity coefficient in different spectral signals. These methods were as follows: absorptivity centering method (a-Centering), absorbance subtraction method (AS), amplitude modulation method (AM,) and amplitude summation method (A-Sum). These methods were applied to determine the binary mixture of ofloxacin (OFX) and dexamethasone (DXM). Linearity of the proposed methods was investigated in the range of 1.0–10.0 *μ*g/ml for both drugs. The proposed methods were validated as per ICH guidelines and were successfully applied for the simultaneous determination of OFX and DXM in their pharmaceutical preparation without interference from additives. Statistical analysis of the results obtained by the proposed spectrophotometric methods compared with a reported method revealed no significant difference between the proposed and reported methods, confirming accuracy and precision at 95% confidence limit.

## 1. Introduction

Through this investigation, the proposed methods were applied to the binary mixture of ofloxacin (OFX) and dexamethasone (DXM) present in eye drop preparation. Ofloxacin (OFX) [9-fluoro-2, 3-dihydro-3-methyl-10-(4-methyl-1-piperazinyl)-7-oxo-7H-pyrido [1,2,3-de]-1, 4-benzoxazine-6-carboxylic acid] is a fluoroquinolone antibacterial agent which is active against wide range of bacteria, whereas dexamethasone (DXM) [9*α*-fluoro-11*β*, 17*α*, 21-trihydroxy-16*α*-methylpregna-1, 4-diene-3, 20-dione] is a corticosteroid with potent anti-inflammatory and immunosuppressive effects [1, 2]. By reviewing literature, several methods reported were reported for the determination of OFX, such as titrimetry [3], spectrophotometry [4, 5], and chromatography [6, 7]. For DXM, reported methods were found, such as spectrophotometry [8, 9], micellar liquid chromatography [10], HPLC [11, 12], and capillary electrophoresis [13]. Simultaneous determination of ofloxacin and dexamethasone phosphate has been reported in the literature by spectrophotometric methods [14, 15] and chromatographic methods [16, 17].

The resolution and analysis of mixtures is an everyday challenge for analysts. Researchers in the analytical field have to come up with new ideas to solve different problems faced by analysts, such as the availability of apparatus, sensitivity, selectivity, cost, environmental aspects, and so on. Spectrophotometry has many advantages for analysts, as they are easy to operate, of low cost, available, and environment-friendly. Fortunately, the last era showed a remarkable development in the invention and application of smart spectrophotometric solutions. In this work, investigation of the efficiency power of recently introduced spectrophotometric methods, which are based on different functions of the absorptivity coefficient, was done.

The term “isosbestic point” describes the intersection point of spectra of prepared mixtures to obtain the sum of the concentrations of the absorbing components in the binary mixture. The iso-absorptive point is defined as the isosbestic point in zero-order absorption spectra where the two absorbing components, *X* and *Y*, have equal absorptivity coefficient [18, 19]. The iso-absorptive point of an absorption spectrum will be retained at the same point in the corresponding ratio spectrum, but it will be shifted to a different adjacent point in the corresponding derivative spectrum [20].

The function of iso-absorptive points has been utilized for different purposes, such as the estimation of a precursor fraction in a complex or conversion reaction [21–23], the direct calculation of concentration of single component using official methods [3], the study of dissolution rate of certain drugs with no inference of excipients [24], and determining the concentrations of several components in pharmaceutical mixtures [20, 25–27].

The aim of the work was to validate the four proposed methods via ICH guidelines, as well as to investigate the efficiency power of four spectrophotometric methods based on iso-absorptivity coefficient point and its manipulated spectra either ratio, using the interfering substance as a divisor or derivative. Absorptivity centering method (a-Centering) and absorbance subtraction method (AS) were applied to zero-order spectra, whereas amplitude modulation method (AM) was applied to ratio spectra and amplitude summation method (A-Sum) was applied to derivative spectra.

### 1.1. Theoretical Background of Absorptivity Centering Method (a-Centering)

This novel method [28] is applied to a mixture of two components (*X* and *Y*) having partially or completely overlapped spectra and showing an intersection at the iso-absorptive point (*λ*_iso_). For the binary mixture of *X* and *Y* showing overlapped spectra (*λ*_iso_) and *X* showing no contribution at (*λ*_2_), this method can be explained through the following five steps.

#### 1.1.1. Calculating the Absorbance of *Y* at (*λ*_iso_) via Absorptivity Factor [*a*_*λ*_iso__/*a*_*Y*(*λ*_2_)_]

*X* and *Y* show overlapped spectra at (*λ*_iso_), whereas *Y* exhibits absorbance at (*λ*_2_), with no contribution of *X*. So, the absorptivity factor is calculated by selecting these two wavelengths (*λ*_iso_ and *λ*_2_) at zero-order absorption spectra (*D*_0_) of different concentrations of pure *Y*; then, the average value is calculated as follows:

(1) $$\begin{array}{c} \frac{A_{Y\left(\lambda_{\text{iso}}\right)}}{A_{Y\left(\lambda_{2}\right)}}=\frac{a_{\lambda_{\text{iso}}}\cdot C_{Y}}{a_{Y\left(\lambda_{2}\right)}\cdot C_{Y}}=\frac{a_{\lambda_{\text{iso}}}}{a_{Y\left(\lambda_{2}\right)}}. \end{array}$$

By multiplying the absorbance (*A*_*λ*_2__), which is the absorbance of the mixture of (*X* + *Y*) at *λ*_2_, by the previously calculated absorptivity factor *a*_*λ*_iso__/*a*_*Y*(*λ*_2_)_  , the absorbance of *Y* in the mixture at *λ*_iso_ [*A*_*Y*(*λ*_iso_)  _] is calculated as shown by the following equation:

(2) $$\begin{array}{c} A_{Y\left(\lambda_{\text{iso}}\right)}\;\;=\frac{\;\;a_{\lambda_{\text{iso}}}}{a_{Y\left(\lambda_{2}\right)}}\cdot A_{\text{mix}\left(\lambda_{2}\right)}. \end{array}$$

#### 1.1.2. Calculating the Absorptivity Inverse at Iso-Absorptive Point (1/*a*_*λ*_iso__)

The zero-order absorption spectra (*D*_0_) of different concentrations of pure *Y* at the selected wavelength (*λ*_iso_) are recorded. Each concentration (*C*) is divided by its corresponding absorbance at the iso-absorptive point *A*_*Y*(*λ*_iso_)_, and then, the average of these values is calculated. This can be summarized by the following equations:

(3) $$\begin{array}{c} \frac{C_{Y}}{A_{Y\left(\lambda_{\text{iso}}\right)}}=\frac{C_{Y}}{a_{Y\;\;\left(\lambda_{\text{iso}}\right)}\cdot C_{Y}}. \end{array}$$

At iso-absorptive point (*λ*_iso_), *a*_*Y*(*λ*_iso_)_  =*a*_*X*(*λ*_iso_)_  =*a*(*λ*_iso_); thus,

(4) $$\begin{array}{c} \frac{C_{Y}}{a_{Y\left(\lambda_{\text{iso}}\right)}\cdot C_{Y}}=\frac{1}{a\;\;\left(\lambda_{\text{iso}}\right)}. \end{array}$$

#### 1.1.3. Preparation of Normalized and Factorized Spectra of *Y*

The normalized spectrum (NS′) represents the absorptivity values of *Y* over the selected wavelength range. It is prepared by dividing each of the zero-order absorption spectra (*D*_0_) of *Y* by its corresponding concentration, and the average value is recorded:

(5) $$\begin{array}{c} \text{normalized spectrum}\;\;\left({\text{NS}}^{\prime }\right)=\frac{a_{Y}\cdot C_{Y}}{C_{Y}}=a_{Y}. \end{array}$$

The factorized spectrum (FS′) represents a spectrum with unity absorbance at the iso-absorptive point [*A*_*Y*(*λ*_iso_)_=1]. It is prepared by dividing each of the zero-order absorption spectra (*D*_0_) of *Y* by its corresponding absorbance at the iso-absorptive point *A*_*Y*  (*λ*_iso_)_, and the average value is recorded:

(6) $$\begin{array}{c} \text{factorized spectrum}\;\;\left({\text{FS}}^{\prime }\right)=\frac{A_{Y}}{A_{Y\left(\lambda_{\text{iso}}\right)}}=\frac{a_{Y}\cdot \;\;C_{Y}}{a_{Y\left(\lambda_{\text{iso}}\right)}\cdot \;\;C_{Y}}=\frac{a_{Y}}{a_{Y\left(\lambda_{\text{iso}}\right)}}. \end{array}$$

#### 1.1.4. Spectrum Recovery of *Y* via Normalized and Factorized Spectra

To recover the zero-order absorption spectrum (*D*_0_) of *Y* via the normalized spectrum (NS′), the absorbance of *Y* at *λ*_iso_*A*_*Y*(*λ*_iso_)_ is multiplied by the calculated absorptivity inverse 1/*a*(*λ*_iso_); then, the result is multiplied by the normalized spectrum of *Y* (*a*_*Y*_):

(7) $$\begin{array}{c} D_{0}\text{ spectrum of}\;\;Y=\;\;A_{Y\left(\lambda_{\text{iso}}\right)}\cdot \frac{1}{a\;\;\left(\lambda_{\text{iso}}\right)}\cdot \left({\text{NS}}^{\prime }\right)=a\left(\lambda_{\text{iso}}\right)\cdot \;\;C_{Y}\cdot \frac{1}{a\;\;\left(\lambda_{\text{iso}}\right)}\cdot a_{Y}=a_{Y}\cdot C_{Y}. \end{array}$$

On the other hand, the absorbance of *Y* at *λ*_iso_*A*_*Y*(*λ*_iso_)_ is directly multiplied by the factorized spectrum (FS′) to recover the zero-order absorption spectrum (*D*_0_) of *Y*:

(8) $$\begin{array}{c} D_{0}\text{ spectrum of}\;\;Y=A_{Y\left(\lambda_{\text{iso}}\right)}\cdot \left({\text{FS}}^{\prime }\right)=a\left(\lambda_{\text{iso}}\right)\cdot \;\;C_{Y}\cdot \frac{a_{Y}}{a\;\;\left(\lambda_{\text{iso}}\right)}=a_{Y}\cdot C_{Y}. \end{array}$$

#### 1.1.5. Spectrum Subtraction to Recover *X* Spectrum

Samir et al. [29] previously introduced this complementary step. By subtracting the previously obtained *D*_0_ spectra of *Y* from the spectra of the mixtures (*X* + *Y*), the *D*_0_ spectra of *X* will be recovered. The concentration of *X* and *Y* is calculated from the corresponding regression equations obtained by plotting the absorbance values of the zero-order curves of *X* or *Y* at its *λ*_max_ against the corresponding concentrations.

## 2. Experimental

### 2.1. Instrument and Chemicals

Spectrophotometer UV-Visible double beam (Shimadzu - UV 1800, Nakagyo-ku, Kyoto, Japan) with matched 1 cm quartz cells operated by Shimadzu UV-Probe 2.32 system software. A solvent mixture was prepared using ethanol HPLC grade (S D Fine-Chem Limited, Mumbai) and distilled water in the ratio of 50 : 50 v/v.

### 2.2. Standard Solutions

Pure samples of ofloxacin (OFX), dexamethasone (DXM), and benzalkonium chloride (BNZ) were kindly supplied by Egyptian International Pharmaceutical Industries Co. (EIPICO), 10^th^ Ramadan city, Cairo, Egypt. The purity was checked by the official methods [3], and it was found to be 100.33 ± 0.73 and 100.51 ± 0.83 for OFX and DXM, respectively. Dexaflox® eye drops (batch number: LB060) was manufactured by Jamjoom Pharma, Jeddah, Kingdom of Saudi Arabia (KSA). Each 1 ml is labeled to contain OFX (3 mg), DXM (1 mg), and BNZ (0.06 mg). Stock standard solutions of OFX, DXM, and BNZ were prepared of concentration 1.0 mg/ml, and further dilution was done using the previously prepared solvent mixture to reach a concentration of 20.0 *μ*g/ml. of each component.

### 2.3. Spectral Manipulations

Solutions equivalent to 1.0–10.0 *μ*g/mL of OFX and DXM were prepared separately in the solvent mixture, and the absorption spectra of the prepared solutions were measured at (200–400 nm) and stored in the computer. The calibration curves were constructed by plotting the absorbance of the zero-order absorption spectra (*D*_0_) of OFX and DXM at 298 and 239 nm, respectively, against the corresponding concentrations, and the linear regression equations were computed. The absorptivity factor of OFX was calculated at the wavelengths [*a*_238_/*a*_298_] by dividing the absorbance of different concentrations of pure OFX at 238 nm/298 nm. The normalized spectrum (NS′) is prepared by dividing difference (*D*_0_) of OFX by its corresponding concentration. The factorized spectrum (FS′) is prepared by dividing the difference (*D*_0_) of OFX by its corresponding absorbance at 238 nm. The absorptivity inverse at iso-absorptive point (1/*a*_238nm_) for OFX is calculated by dividing the zero-order absorption spectra (*D*_0_) of OFX by its normalized spectrum (NS′) to get the spectrum representing the concentration; then, the obtained spectrum is divided by its corresponding zero-order spectrum to get that spectrum absorptivity inverse (1/*a*) all over the wavelength range. Thus, the recorded value at the iso-absorptive point (238 nm) is equal to absorptivity inverse (1/*a*_238nm_). The average values for the constants were calculated. The stored absorption spectra of OFX and DXM were separately divided by the normalized OFX spectrum, and the obtained ratio spectra were recorded. Calibration curves were constructed by plotting both of their amplitudes at *λ*_iso_ 238.0 nm against their corresponding concentrations, and the regression equations were computed. Calibration curves were constructed at *λ*_max_ for both drugs at 298.0 nm and 239.5 nm for OFX and DXM, respectively, against their corresponding concentrations, and the regression equations were computed. The zero-order absorption spectra were derivatized in first order using ∆*λ*_8_ and scaling factor 10 and the first derivative amplitudes *D*_1_ were recorded. The calibration curves were constructed by plotting the amplitudes of OFX and DXM at 246.5 nm against their corresponding concentrations, and the regression equations were computed. The amplitude factor of OFX was calculated at these selected wavelengths [*D*_246.5_/*D*_307_] by dividing the *D*_1_ amplitudes of different concentration of pure OFX at 246.5 nm/307 nm, and the average value of each was calculated.

### 2.4. Application to Synthetic Mixtures and Pharmaceutical Preparation

Into a series of 10 mL volumetric flasks, accurate aliquots of OFX and DXM were transferred from their working solutions (20.0 *μ*g/ml) to prepare nine mixtures containing different ratios of the cited drugs. Aliquot equivalent to 1 *μ*g/mL of BNZ is added to each prepared mixture. The volumes were completed with the solvent mixture. The spectra of the prepared solutions from 200 to 400 nm were recorded and stored. One millimeter of Dexaflox® eye drops was transferred into 50 mL volumetric flask, and the prepared solution was filtered through 0.45 *μ*m Millipore syringe membrane filter. An appropriate dilution was made with the same solvent mixture to prepare the working solution claimed to contain 9.0 *μ*g/ml OFX and 3.0 *μ*g/ml DXM. Six replicates for each experiment were done. The concentrations of pure OFX and DXM were calculated using their corresponding regression equations.

## 3. Results and Discussion

**Figure 1:**
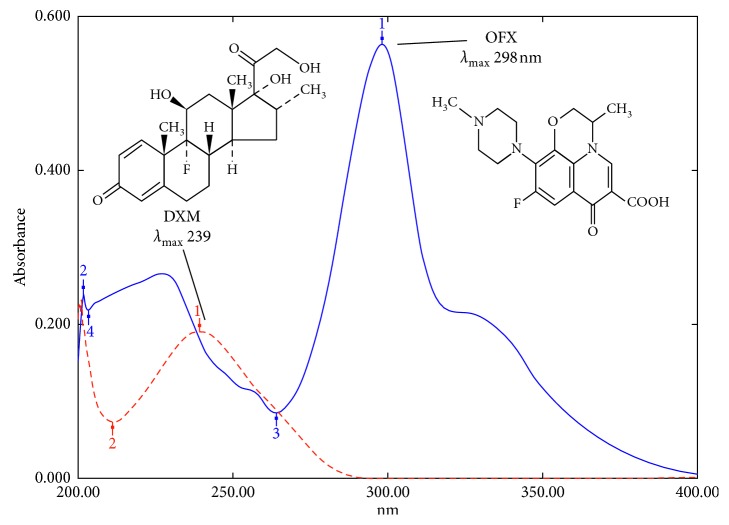
The zero-order spectra (*D*_0_) and chemical structures of OFX and DXM showing *λ*_max_.

These methods were applied to the binary mixture of ofloxacin (OFX) and dexamethasone (DXM). The zero-order spectra (*D*_0_) and chemical structures for the cited drugs are shown in Figure 1, where OFX and DXM show overlapped spectra at (*λ*_iso_ 238 nm), but OFX exhibits absorbance at 298 nm solely with no interference with DXM. The solvent mixture was prepared in that ratio in order to reduce the use of organic solvent and cost and have less impact on the environment.

### 3.1. Absorptivity Centering Method (a-Centering)

This method starts with the calculation of two constants for OFX: the absorptivity factor [*a*_238_/*a*_298_] and the absorptivity inverse at the iso-absorptive point (1/*a*  _238_), which were found to be equal to 0.339 and 25.833, respectively. The absorbance of OFX in mixtures' spectra at the iso-absorptive point *λ*_iso_ “*A*_(238nm)_” was calculated by multiplying the absorbance of OFX at 298.0 nm by the calculated absorptivity factor [*a*_238_/*a*_298_], as shown in the equation:

(9) $$\begin{array}{c} A_{\text{OFX}\left(238\right)}\;\;=\frac{a_{238\;\;}}{a_{298}}\cdot A_{\text{mix}\left(298\right)}. \end{array}$$

**Figure 2:**
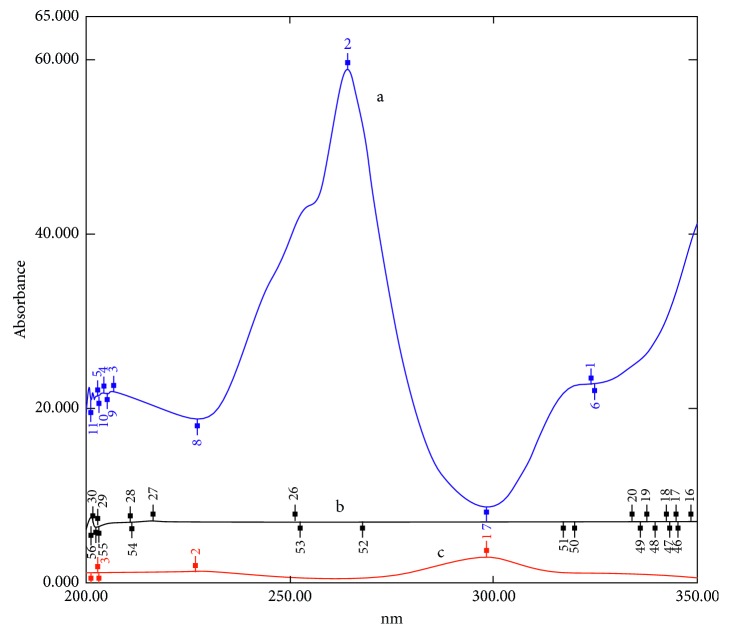
OFX spectra representing (a) absorptivity inverse (1/*a*), (b) concentration of OFX in mixture 8 *μ*g/ml, and (c) factorized spectrum of OFX (FS′).

Two approaches could be adopted in order to recover the zero-order absorption spectra *D*_0_ of OFX from the *D*_0_ spectra of the mixtures. The first approach was done by multiplying the calculated absorbance of OFX (*A*_238_) by the calculated absorptivity inverse (1/*a*  _238_), and then the resulting spectrum is multiplied by the normalized spectrum (NS′) of OFX, as shown in Figure 2. The second approach was done by multiplying the calculated absorbance of OFX (*A*_238_) by the prepared factorized spectrum (FS′) of OFX directly, as shown in Figure 2.

The zero-order absorption spectra *D*_0_ of DXM can be recovered by subtracting the recovered spectrum of OFX from the from the mixtures' spectra. The concentration of OFX and DXM is calculated from the corresponding regression equations at their *λ*_max_ 298.0 and 239.5 nm, respectively, as shown in Figure 1.

By putting this method in hand, it was found that the approach via factorized spectrum (FS′) is much simpler than that of the normalized spectrum (NS′). In spite of the several preparatory steps involved in this method (especially using the NS′ approach), its advantage not only lies in the calculation of each component's concentration at its *λ*_max_, which ensures its accuracy and sensitivity, but also in the simplicity of recovering the whole spectrum of each component, which acts as a spectral profile for it, and can be further used in testing its purity [30].

### 3.2. Absorbance Subtraction Method (AS)

**Figure 3:**
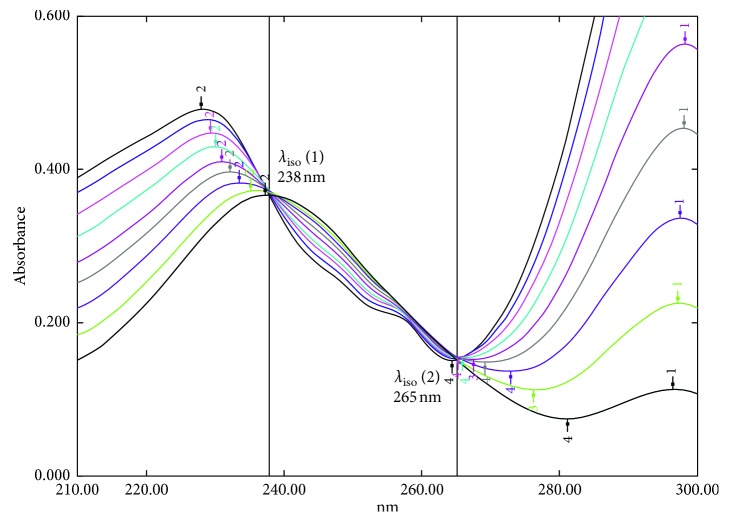
The zero-order spectra (*D*_0_) of mixtures of (OFX + DXM) with a total concentration equal to (10 *μ*g/ml) showing 2 iso-absorptive points.

The binary mixture of OFX and DXM exhibits two iso-absorptive points (*λ*_iso_) at 238 and 265 nm where the components exhibiting this point have equal absorptivity coefficient, as shown in Figure 3. The calculation was done at the first point (*λ*_iso_) 238 nm because it has higher absorbance signal which increases accuracy and sensitivity of the results. The absorbance of OFX at *λ*_iso_ “*A*_(238nm)_” was calculated using the calculated absorptivity factor [*a*_238_/*a*_298_] as explained before in the absorptivity centering method; then, the absorbance of DXM *A*_DXM(238)_ was obtained by subtracting the absorbance of OFX from the absorbance of the mixture *A*_mix(238)_. The absorbance values of OFX and DXM at *λ*_iso_ 238 nm were used to calculate each of their concentration using the unified regression equation at the same wavelength.

(10) $$\begin{array}{c} A_{\text{DXM}\left(238\right)}\;\;=A_{\text{mix}\left(298\right)}-A{\;\;}_{\text{OFX}\left(238\right)}. \end{array}$$

This method exceeds in simplicity as it does not require any manipulating steps except for the factor calculation and both components can be determined using unified regression equation at *λ*_iso_. The disadvantage of this method is measuring the absorbance at wavelength rather than *λ*_max_, which increases the risk of error when calculating low concentrations, in addition to the inability of testing purity by extracting the whole spectrum (as in absorptivity centering method).

### 3.3. Amplitude Modulation Method (AM)

**Figure 4:**
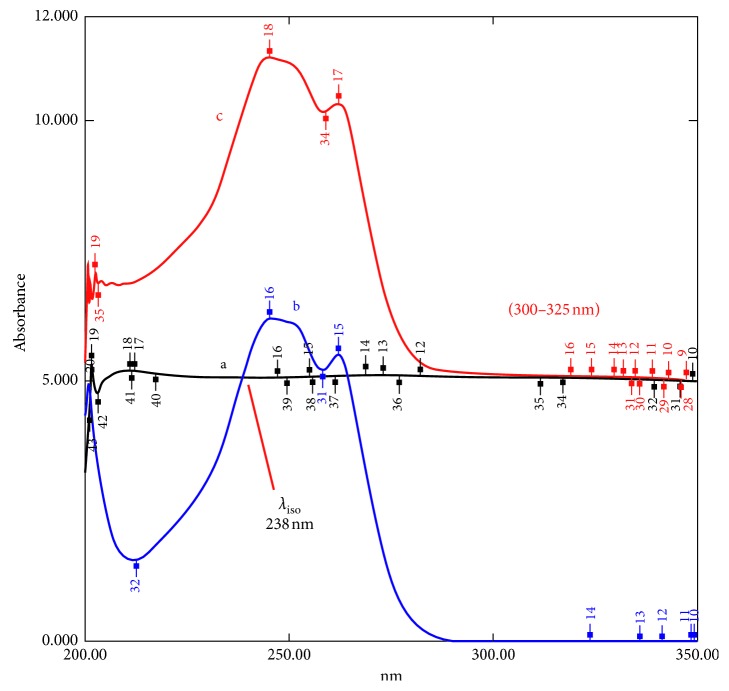
The ratio spectra of (a) OFX (5 *μ*g/ml), (b) DXM (5 *μ*g/ml), and (c) a mixture of OFX + DXM (5 *μ*g/ml each), using normalized spectrum (NS′) of OFX as a divisor.

This manipulating ratio-spectra technique depends on the fact that the iso-absorptive point in a zero-order absorption spectrum (*D*_0_) will be retained in the ratio spectrum at the same point. The zero-order absorption spectrum of OFX is extended over that of DXM. To eliminate the effect of the divisor choice, the normalized spectra (NS′) of OFX was used as a divisor. When the *D*_0_ spectrum of the binary mixture was divided by the (NS′) OFX divisor, a ratio spectrum with a constant amplitude value at the plateau region at (300–325 nm) was obtained, as shown in Figure 4. The recorded constant amplitude at this plateau region was modulated to be directly equal to the concentration of OFX (*C*_Recorded of OFX_):

(11) $$\begin{array}{c} P_{\text{mix}}=P_{\text{DXM}}+P_{\text{OFX}},\\ P_{\text{mix}}=\frac{a_{\text{DXM}}\cdot C_{\text{DXM}}}{a_{\text{OFX}}}\;\;+\frac{a_{\text{OFX}}\cdot C_{\text{OFX}}}{a_{\text{OFX}}},\\ P_{\text{mix}}=\;\;\frac{a_{\text{DXM}}\cdot C_{\text{DXM}}}{a_{\text{OFX}}}+\text{constant},\\ P_{\text{OFX}}\left(\text{constant}\right)=\frac{a_{\text{OFX}}\cdot C_{\text{OFX}}}{a_{\text{OFX}}}=C_{\text{OFX}}, \end{array}$$

where *P*_mix_ is the *P* amplitude value of the binary mixture, *P*_OFX_ and *P*_DXM_ are the ratio amplitudes of OFX and DXM, respectively, “*a*” is the absorptivity coefficient, and “*C*” is the concentration.

On the other hand, the amplitude at the *λ*_iso_ at 238 nm was equal to the sum of the concentrations of OFX and DXM. By subtracting the previously obtained constant from the recorded amplitude at *λ*_iso_, the obtained amplitude was directly modulated to the concentration of DXM in the mixture (*C*_Recorded of DXM_):

(12) $$\begin{array}{c} P_{\text{mix}}=P_{\text{DXM}}+\text{ constant,}\\ P_{\text{DXM}}=\;\;\left[\;\;\frac{a_{\text{DXM}}\cdot C_{\text{DXM}}}{a_{\text{OFX}}}+\text{constant}\right]-\text{constant},\\ P_{\text{DXM}}\;\;=\;\;\frac{a_{\text{DXM}}\cdot C_{\text{DXM}}\;\;}{a_{\text{OFX}}},\\ ∵\text{ at}\;\;\lambda_{\text{iso}}\text{:}\;\;a_{\text{DXM}}\;\;=a_{\text{OFX}},\\ ∴\;\;P_{\text{DXM}}\;\;=C_{\text{DXM}}. \end{array}$$

One regression equation is constructed for both drugs at the iso-absorptive point (*λ*_iso_ 238 nm), where its slope is almost one and its intercept is almost zero. The equation is used to eliminate the signal to noise ratio for both OFX and DXM.

The amplitude modulation (AM) method excels over other manipulating ratio-spectra techniques by reducing the manipulation steps and elimination of the choice of the divisor. Only one divisor is needed to determine both components in the mixture and directly modulate the amplitudes into concentrations.

### 3.4. Amplitude Summation Method (A-Sum)

**Figure 5:**
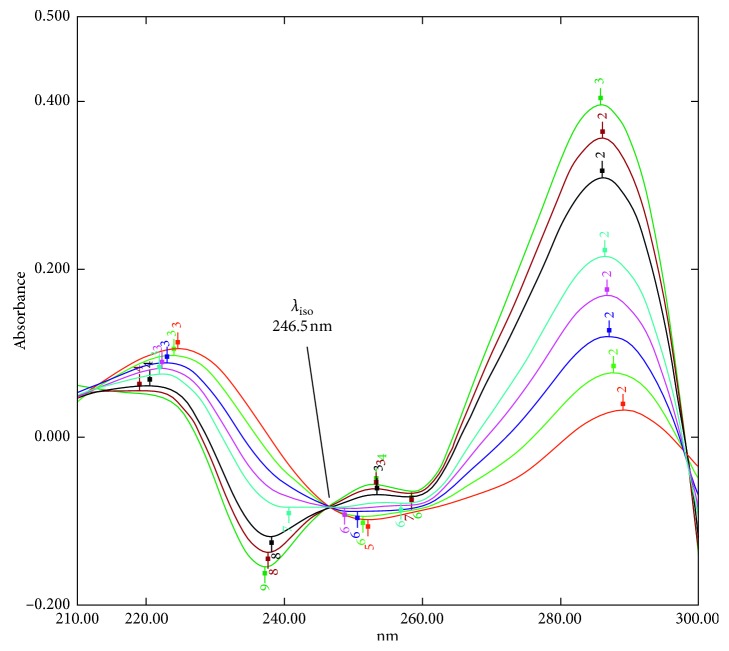
The first derivative spectra (*D*_1_) of mixtures of (OFX + DXM) with a total concentration equal to 10 *μ*g/ml showing isosbestic point at 246.5 nm.

The spectrum of OFX and DXM mixture showed one isosbestic point (*λ*_iso_) only at 246.5 nm, as shown in Figure 5, where OFX shows overlap with DXM, whereas at 307.0 nm, DXM shows no contribution. The *D*_1_ amplitude of OFX in the mixtures at (*λ*_iso_) 246.5 nm was calculated by multiplying the *D*_1_ amplitude of the mixture at 307.0 nm by its amplitude factor [*D*_246.5_/*D*_307_]. Then, the amplitude of DXM at (*λ*_iso_) 246.5 nm in the mixtures was obtained by subtracting the calculated amplitude of OFX at the same point. The *D*_1_ amplitude of OFX and DXM was used to calculate each of their concentration using the unified regression equation at 246.5 nm:

(13) $$\begin{array}{c} D_{\text{OFX}\left(246.5\right)}=\frac{D_{246.5}}{D_{307}}\cdot D_{\text{mix}\left(307\right)},\\ D_{\text{DXM}\left(246.5\right)}=D_{\text{mix}\left(246.5\right)}–D_{\text{OFX}\left(246.5\right)}, \end{array}$$

where *D*_mix_ is the *D*_1_ amplitude value of the binary mixture, *D*_OFX_ and *D*_DXM_ are the *D*_1_ amplitudes of OFX and DXM, respectively, and [*D*_246.5_/*D*_307_] is the amplitude factor of OFX.

Few differences were observed when applying this method: The shift that occurred for the isosbestic point (from 238.0 to 246.5 nm) is due to derivatization manipulation Δ*λ* = 8 and scaling factor 10. The main advantage in this method is that there is no need for a divisor and the sensitivity of the method is enhanced when compared with zero-order absorption spectra. In addition, the shift that occurred in the iso-point gives an opportunity for more complex mixtures to correct any interference of any component that may possesses a zero derivative value at the isosbestic point.

A brief comparison between the four methods, including the strength and weakness points, is summarized in Table 1. Overall, the absorptivity centering method (a-Centering) via normalized spectrum involves several manipulating steps, so it is more convenient when applied via factorized spectrum. Both approaches are recommended to be used in case of recovering the full spectrum of each component with maximum accuracy and precision, as measurements are done at *λ*_max_ of each component. On the other hand, the three remaining methods determine both components via unified regression equation. The absorbance subtraction (AS) method is considered to be the simplest method with minimum manipulation but suffers from inaccuracy in case of low concentration of the extended drug which obstacles the calculation of the absorbance factor. The amplitude modulation (AM) method is considered to be the only method which can directly modulate amplitudes into concentrations without need for standard calibration curves. Finally, the amplitude summation (A-Sum) method becomes of use when shifting the isosbestic point is needed to eliminate interference from excipients or other components.

### 3.5. Validation, System Suitability, and Statistical Analysis

The validation parameters for the proposed methods were applied as per the ICH guidelines [31], including linearity, range, accuracy, precision, and system suitability. Linear regression equations were constructed by plotting the concentration (within the selected range) as an independent variable (*X*) versus the corresponding absorbance as an independent variable (*Y*) to apply a best fit straight regression line. The precision study included the triplicate measurements of three concentrations of each drug which were done on the same day (repeatability) and on three successive days (interday precision). Satisfactory results were obtained for all validation parameters, as shown in Table 2. The accuracy of the proposed methods was assessed by the analysis of synthetic mixtures containing different ratios of the cited drugs, as shown in Table 3.

System suitability [32] was measured for standard solutions containing 100% working concentration for six replicates, and the %RSD was calculated, as shown in Table 2. The proposed methods were applied for the determination of Dexaflox® eye drops and the validity of the proposed procedures is further assessed by comparing it with a reported method [14]. The results obtained are shown in Table 4.

Statistical analysis testing (one-way *ANOVA*) was applied for the purpose of comparison between the recovery percent obtained by the analysis of the synthetic mixtures by the proposed methods; Table 5 shows that there was no significant difference between them.

## 4. Conclusion

This work introduced an investigation concerning the efficiency of four methods utilizing different functions of absorptivity coefficient in different absorption spectra: absorptivity centering method (a-Centering), absorbance subtraction method (AS), amplitude modulation method (AM), and amplitude summation method (A-Sum). In conclusion, the absorptivity centering method was found to have the optimum efficiency power to resolve the unresolved spectra and recovering of their zero-order absorption spectra which acts as their own fingerprint and reflect the purity of the drugs. Thus, OFX and DXM can be directly determined by the suggested methods in pure and pharmaceutical preparations without introductory separation. Additionally, these methods do not need sophisticated techniques, instruments, or high-priced solvents or a harmful organic solvent. Consequently, they can be used for the routine analysis of cited drugs in laboratories lacking liquid chromatographic instruments.

## Figures and Tables

**Table 1:** A brief about the investigation of the four applied spectrophotometric methods.

Comparison points	a-Centering	AS	AM	A-Sum
Spectral signal	Zero-order	Zero-order	Ratio	Derivative

Number *λ* of measurements (for both drugs)	2 *λ*_max(*x*)_, *λ*_max(*y*)_	1 *λ*_iso_	1 *λ*_iso_	1 *λ*_iso_

Prepared factors	(NS′): *a*_*λ*_iso__/*a*_*Y*(*λ*_2_)_+1/*a*_*λ*_iso__ (FS′): *a*_*λ*_iso__/*a*_*Y*(*λ*_2_)_	*a*_*λ*_iso__/*a*_*Y*(*λ*_2_)_	None	*D*_*λ*_iso__/*D*_*λ*_2__

Prepared spectra	(NS′)/(FS′)	None	(NS′) divisor	None

Number of manipulating steps	(NS′): 4 (FS′): 3	2	3	3

Advantages	(i) Max accuracy (*λ*_max_)	(i) Simplicity	(i) Direct modulation of amplitude to concentration	(i) Maximum sensitivity
	(ii) Testing purity	(ii) Minimum manipulation		(ii) Eliminate interference

NS′: normalized spectrum; FS′: factorized spectrum.

**Table 2:** Assay parameters and validation sheet obtained by applying the proposed spectrophotometric methods.

Parameters	a-Centering		AS		AM		A-Sum	
	OFX	DXM	OFX	DXM	OFX	DXM	OFX	DXM
Wavelength (nm)	*D*_0_ at 298	*D*_0_ at 239.5	*D*_0_ at 238		*P* at 238 nm		*D*_1_ at 246.5 nm	
Calibration range^a^ (*µ*g/mL)	1–10	3–10	3–10		3–10		2–10	
Slope	0.1154	0.0382	0.0387		1.0012		0.0087	
Intercept	−0.0084	−0.0008	−0.0008		−0.0011		−0.0017	
Correlation coefficient (*r*)	0.9999	0.9998	0.9998		0.9999		0.9999	
Mean^a^	99.90	99.52	100.17		99.89		100.53	
RSD%	0.666	0.515	0.931		0.449		0.516	
Accuracy^ab^	100.78 ± 1.061	99.97 ± 0.687	100.32 ± 1.002	99.55 ± 0874	99.32 ± 0.417	100.11 ± 0.744	100.54 ± 0.987	99.88 ± 0.552
Repeatability^ac^	±0.56/0.559	±0.68/0.684	±0.47/0.474	±0.96/0.965	±0.63/0.633	±0.41/0.411	±0.54/0.541	±0.97/0.965
Interday precision^ac^	±0.70/0.698	±0.71/0.711	±0.55/0.552	±1.11/1.105	±0.73/0.729	±0.63/0.632	±0.71/0.711	±0.97/0.977
Robustness^acd^	100.06/0.785	99.23/0.884	100.33/0.877	99.52/0.987	99.99/0.719	100.98/1.050	100.41/0.963	99.87/1.114
System suitability RSD%^e^	0.774	0.574	0.411	0.217	0.419	0.622	0.796	0.433

^a^Average of three experiments. ^b^Mean ± standard deviation of 3 concentrations of each drug (4, 6, and 8 *µ*g/mL). ^c^Standard deviation/relative standard deviation of 3 concentrations of each drug (4, 6, and 8 *µ*g/mL). ^d^Robustness was checked by testing the effect of the solvent (29, 31, and 32% methanol). ^e^OFX and DXM (6 *µ*g/mL).

**Table 3:** Analysis of synthetic mixtures by the proposed spectrophotometric methods.

OFX : DXM (*µ*g/mL)^a^	a-Centering		AS		AM		A-Sum	
	OFX	DXM	OFX	DXM	OFX	DXM	OFX	DXM
4 : 6	100.00	102.10	100.99	99.12	101.80	99.18	100.87	101.01
4 : 8	101.51	98.35	100.99	100.30	100.56	98.16	98.66	99.39
4 : 12	100.94	100.10	101.77	100.89	101.31	99.67	99.35	99.87
5 : 5	100.52	101.72	100.93	101.62	99.42	98.30	99.89	102.03
6 : 4	101.64	99.26	98.03	100.88	99.31	101.26	102.51	100.88
8 : 6	101.29	100.13	99.31	100.98	98.33	100.22	101.56	101.60
9 : 3^b^	101.78	101.41	102.04	101.74	101.99	99.73	98.96	99.11
Mean	100.17	99.90	100.44	99.82	99.78	100.31	99.66	100.10
±SD	0.825	0.874	0.975	1.228	1.064	1.128	0.664	0.889

^a^Average of three experiments. ^b^Ratio present in Dexaflox® eye drops.

**Table 4:** Application of the proposed and reported methods for the analysis of pharmaceutical preparation.

Methods	OFX		DXM	
	Found (*µ*g/mL)^a^	*F* value^b^	Found (*µ*g/mL)^a^	*F* value^b^
a-Centering	101.52 ± 0.521	1.040	98.52 ± 0.631	0.640
AS	100.56 ± 0.741	2.103	99.63 ± 0.963	1.490
AM	100.23 ± 1.021	3.992	100.32 ± 0.441	0.312
A-Sum	100.85 ± 0.985	3.716	99.78 ± 0.369	0.219
Reported method [14]	99.58 ± 0.511		100.25 ± 0.789	

^a^OFX claimed to be 9 *µ*g/mL, DXM: 3 *µ*g/mL, average of six experiments. ^b^Tabulated *F* value (5.050) at *P* < 0.05.

**Table 5:** One-way ANOVA statistical comparison between the results obtained by the proposed method for the determination of OFX and DXM in bulk form.

Source of variation	Degree of freedom	Sum of squares	Mean square	*F* value^a^	*P* value^a^	*F* critical^a^
OFX						
Between columns	3	2.698	0.8993	1.122	0.7143	2.342
Within columns	24	19.230	0.8012			
Total	27	21.928				

DXM						
Between columns	3	1.013	0.3376	1.699	0.8169	2.342
Within columns	24	26.015	1.084			
Total	27	27.028				

^a^There was no significant difference between the methods using one-way ANOVA at *P* < 0.05.

## Data Availability

The data used to support the findings of this study are included within the article.
